# Anion-Sensitive Fluorophore Identifies the *Drosophila* Swell-Activated Chloride Channel in a Genome-Wide RNA Interference Screen

**DOI:** 10.1371/journal.pone.0046865

**Published:** 2012-10-04

**Authors:** Stephanie C. Stotz, David E. Clapham

**Affiliations:** 1 Howard Hughes Medical Institute, Department of Cardiology, Boston Children’s Hospital, Boston, Massachusetts, United States of America; 2 Manton Center for Orphan Disease, Boston Children’s Hospital, Boston, Massachusetts, United States of America; 3 Department of Neurobiology, Harvard Medical School, Boston, Massachusetts, United States of America; Albany Medical College, United States of America

## Abstract

When cells swell in hypo-osmotic solutions, chloride-selective ion channels (Cl_swell_) activate to reduce intracellular osmolality and prevent catastrophic cell rupture. Despite intensive efforts to assign a molecular identity to the mammalian Cl_swell_ channel, it remains unknown. In an unbiased genome-wide RNA interference (RNAi) screen of *Drosophila* cells stably expressing an anion-sensitive fluorescent indicator, we identify Bestrophin 1 (dBest1) as the *Drosophila* Cl_swell_ channel. Of the 23 screen hits with mammalian homologs and predicted transmembrane domains, only RNAi specifically targeting dBest1 eliminated the Cl_swell_ current (I_Clswell_). We further demonstrate the essential contribution of dBest1 to *Drosophila* I_Clswell_ with the introduction of a human Bestrophin disease-associated mutation (W94C). Overexpression of the W94C construct in *Drosophila* cells significantly reduced the endogenous I_Clswell_. We confirm that exogenous expression of dBest1 alone in human embryonic kidney (HEK293) cells creates a clearly identifiable *Drosophila*–like I_Clswell_. In contrast, activation of mouse Bestrophin 2 (mBest2), the closest mammalian ortholog of dBest1, is swell-insensitive. The first 64 residues of dBest1 conferred swell activation to mBest2. The chimera, however, maintains mBest2-like pore properties, strongly indicating that the Bestrophin protein forms the Cl_swell_ channel itself rather than functioning as an essential auxiliary subunit. dBest1 is an anion channel clearly responsive to swell; this activation depends upon its N-terminus.

## Introduction

All mammalian cells express chloride channels activated by decreases in extracellular osmolality, albeit with different biophysical properties [1]. The ubiquitous expression of Cl_swell_ suggests its essential cellular function. Tightly regulated Cl_swell_ channels participate in volume regulation, motility, cell survival, and cell division [1]. In contrast, de-regulated constitutively active Cl_swell_ channels exacerbate several cardiac diseases, including myocardial hypertrophy and heart failure [2]. The mammalian Cl_swell_ channel- encoding gene has yet to be identified despite the wealth of proteins nominated by candidate approaches [3]. These proteins include ClC-2 [4], ClC-3 [5], P-glycoprotein [6], [7], pICln [8], [9], p64 [10], phospholemman [11], Best1 and 2 [12], TMEM16A [13], and TMEM16F [14]. The research community has yet to agree on any of these candidates as a *bona fide* Cl_swell_ channel.

In *Drosophila*, however, accumulating evidence indicates that *dBest1* encodes for a Cl_swell_ channel. RNAi targeting *dBest1* eliminates *Drosophila* Schneider (S2) cell I_Clswell_, an effect rescued by re-introduction of dBest1 [15]. Further, swell activated dBest1 mutants have altered biophysical properties and reactivity to sulfhydryl reagents [16]. dBest1 likely forms the chloride conducting pore, but it may be an obligate auxillary subunit of *Drosophila* I_Clswell_ that modifies channel properties akin to CaV β subunits [17].

Assigning chloride channel function to any protein is difficult. The known chloride channel families (*e.g.*, ClC, Anoctamin/TMEM16, CFTR, and ionotropic GABA_A_ and GlyR) lack structural pore or gating motifs that might form the basis for *in silico* identification. Expression cloning approaches have also failed due to widespread Cl_swell_ channel expression that precludes the separation of endogenous and over-expressed protein activities. Moreover, known chloride channels blockers are non-specific and their affinities are far too low to encourage affinity purification. Finally, previous chloride indicators are poor tools for screening due to loading and retention issues, inconsistent results, and poor reproducibility [18].

Here we present an unbiased genome-wide, high-throughput RNAi screen designed to identify the *Drosophila* Cl_swell_ channel and its regulators. Our screen employed H148Q-YFP, a genetically encoded anion-sensitive yellow fluorescent protein [19], to report Cl_swell_ activity in *Drosophila* S2R+ cells. Of our 595 initial hits that altered chloride handling, we concentrated on characterizing proteins with mammalian homology and at least one transmembrane domain as potential Cl_swell_ channels. dBest1 emerged from our screen as the lead candidate for *Drosophila* Cl_swell_. Both RNAi knockdown of dBest1 and overexpression of a dominant-negative dBest1 eliminated the Cl_swell_ current in *Drosophila* S2R+ cells. Conversely, dBest1 overexpression in a mammalian system (HEK cells) produced a *Drosophila*–like I_Clswell_. To identify domains necessary for swell activation we characterized chimeras between the swell-sensitive dBest1 and the swell-insensitive mBest2. Swell sensitivity is only apparent in mBest2, the closest mammalian homolog of dBest1, when the protein contains the dBest1 amino (N)-terminus. This chimera maintains the pore properties of mBest2, providing additional evidence that the protein itself forms a channel rather than functioning as a necessary auxiliary subunit. We conclude that dBest1 is the channel underlying the *Drosophila* I_Clswell_.

## Results

### *Drosophila* S2R+ Cells have Robust I_Clswell_

**Figure 1:**
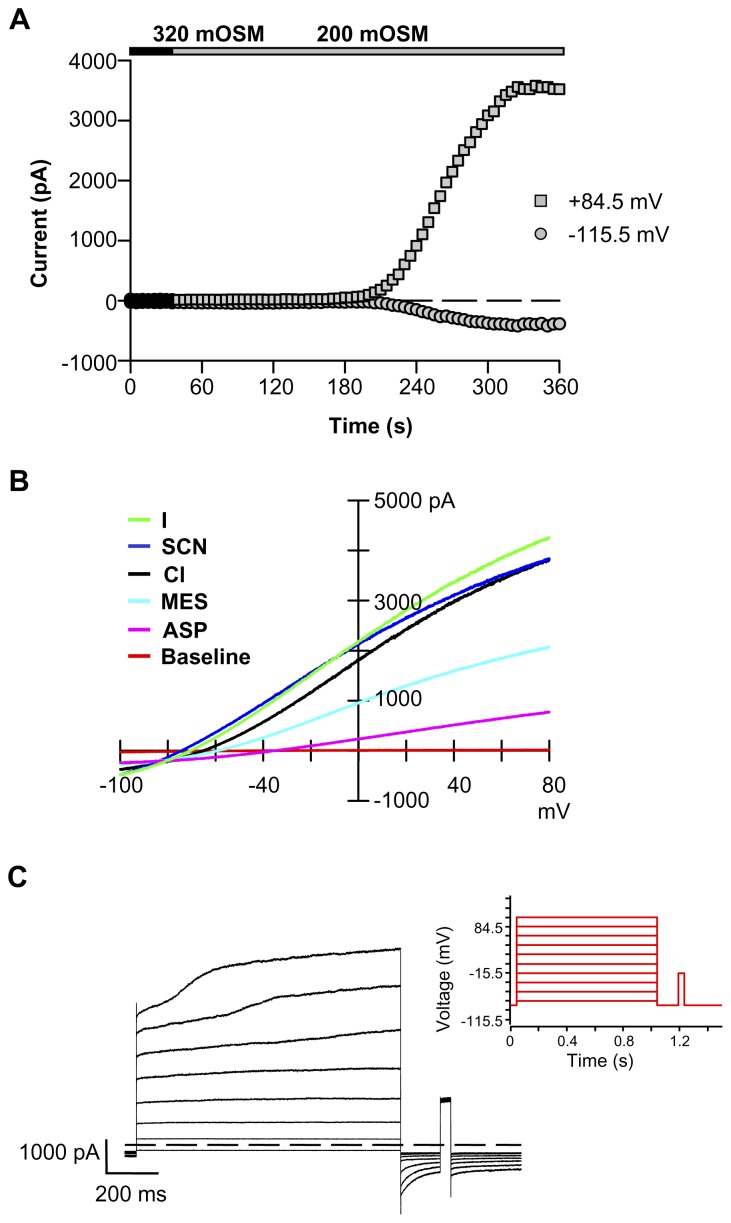
Characterization of the S2R+ cell I_Clswell_. (**A**) Hypo-osmotic solutions slowly activate I_Clswell_. I_Clswell_ begins to activate 1.7±0.3 min after exposure to 200 mOSM solution and reaches steady state activation within 5±0.3 min (n = 13). I_Clswell_ was assessed in ramp protocols and reported at +84.5 mV (upper trace) and -115.5 mV (lower trace). 240 mOSM stimulates I_Clswell_ activation slightly more slowly (1.8±0.3 min to initiation and 5.2±0.6 min to steady state, n = 12; data not shown). (**B**) The S2R+ cell I_Clswell_ is anion-selective. I_Clswell_ was activated by 200 mOSM solution; relative permeability and slope conductance sequences were determined for the steady state I_Clswell_ by replacing Cl^−^ with equimolar anion concentrations. (**C**) An extended step protocol (red inset) reveals more than one set of activation kinetics with offset activation initiation times.

**Figure 2:**
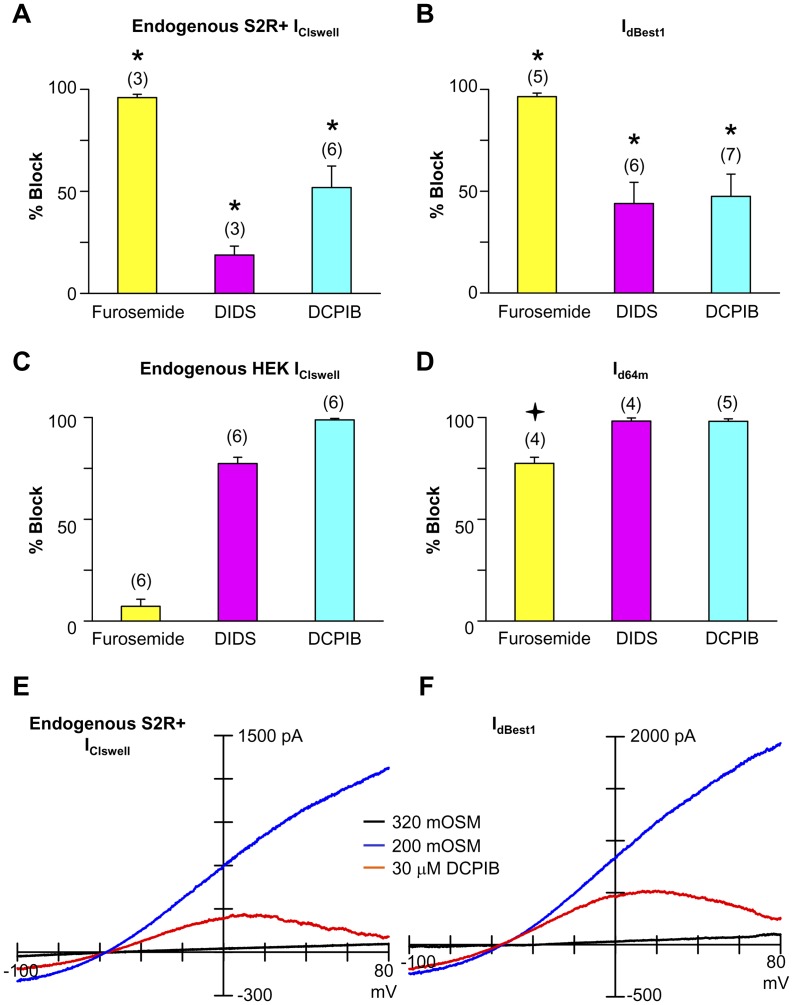
Pharmacological profiles of S2R+ I_Clswell_ and I_dBest1_ match and differ from those of HEK I_Clswell_ and I_d64m_. (A–D) % Block of S2R+ I_Clswell_, I_dBest1_, HEK I_Clswell_ and I_d64m_ by 1 mM furosemide, 100 µM DIDS, and 30 µM DCPIB. Block at 0 mV is presented to emphasize the incomplete voltage-dependent DCPIB block of S2R+ I_Clswell_ and I_dBest1_. (A) Steady state S2R+ I_Clswell_ activated by 200 mOSM stimulation was blocked 96% ±1.6 by furosemide, 19% ±4 by DIDS, and 52% ±10.6 by DCPIB. * no difference compared to I_dBest1_ block and significantly different compared to HEK I_Clswell_ and I_d64m_ (ANOVA, p<0.05). (B) I_dBest1_, stimulated for 2 min by 200 mOSM, was blocked 96% ±1.7 by furosemide, 44% ±10 by DIDS, and 47% ±10.9 by DCPIB. * no difference compared to S2R+ I_Clswell_ block and significantly different compared to HEK I_Clswell_ and I_d64m_ (ANOVA, p<0.05). (C) Steady-state HEK I_Clswell_ activated by 200 mOSM stimulation was blocked 7% ±3.5 by furosemide, 77% ±3 by DIDS, and 99% ±0.7 by DCPIB. (D) Constitutive I_d64m_ (320 mOSM) was blocked 77% ±3 by furosemide, 98% ±1.5 by DIDS, and 98% ±1.2 by DCPIB. 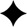 significantly different compared to S2R+ I_Clswell_, HEK I_Clswell_ and I_d64m_ (ANOVA, p<0.05). (E–F) I–V relations for S2R+ I_Clswell_ and I_dBest1_ demonstrate DCPIB voltage-dependent block. At 80 mV, DCPIB block of S2R+ I_Clswell_ is 90% ±3.6 (n = 6), and 82% ±6.5 for I_dBest1_ (n = 7).

**Figure 3:**
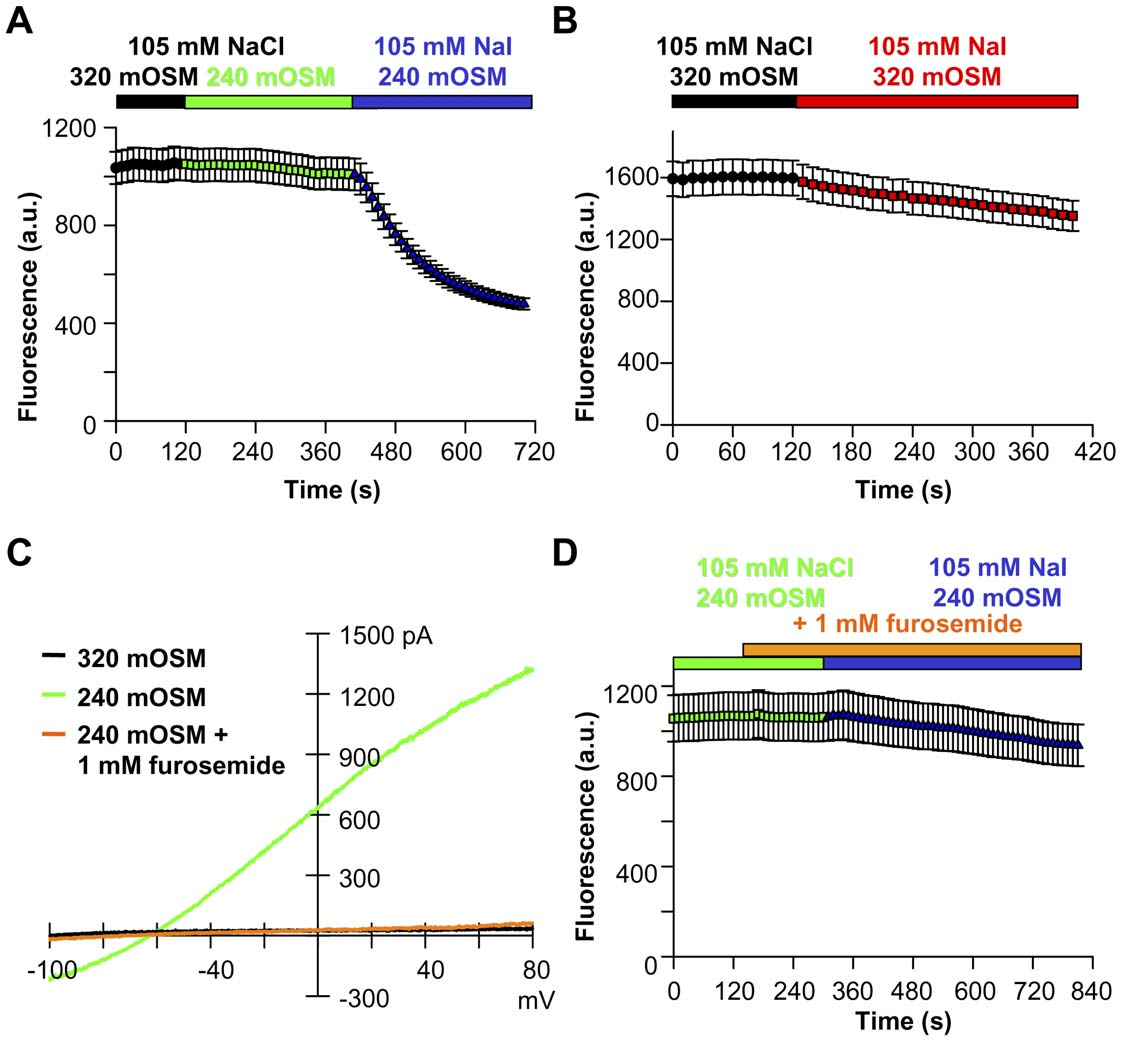
H148Q-YFP stably expressed in S2R+ cells reports the entry of I^−^ through activated Cl_swell_ channels. (**A**) Cellular swelling in 240 mOSM Cl^−^ did not alter fluorescence intensity as Cl_swell_ channels activate (Student’s t-test, p = 0.65; n = 76). Replacement of Cl^−^ with I^−^ evoked a 51% ±1.3 decrease in fluorescence (Student’s t-test, p<0.001; n = 76). Imaging assay; fluorescence is in arbitrary units (a.u.). (**B**) Cl_swell_ channels must be open for I^–^induced fluorescence suppression to occur. 320 mOSM NaI suppresses fluorescence by 16% ±0.7 (Student’s t-test, p = 0.1; n = 54). (**C**) Furosemide, an NKCC2 blocker, completely inhibits the S2R+ cell Cl_swell_ channels at 1 mM (n = 3). (**D**) 1 mM Furosemide block of Cl_swell_ prevents significant I^–^induced suppression of H148Q-YFP fluorescence (Student’s t-test, p = 0.54; n = 16).

*Drosophila* S2 cells are used extensively in genome-wide RNAi screens to dissect signaling pathways, determine protein functions, and assign protein molecular identity [20]. These macrophage-like cells, derived from primary culture of late stage *Drosophila melanogaster* embryos [21] readily take up RNAi from serum-free media. The subsequent process of targeted mRNA ablation is efficient and highly reproducible [22], [23]. For our screen we used S2R+ cells [24], an adherent S2 variant well suited for assays that require multiple solution changes. Importantly, S2R+ cells have a consistent, large I_Clswell_ that activates slowly upon a drop in extracellular osmolality (Figure 1A). I_Clswell_ starts to activate within 2 min exposure to hypo-osmotic media, reaching steady state activation by 5 min. The fully activated Cl_swell_ conductance is anion selective (Figure 1B). The relative permeability sequence of S2R+ Cl_swell_ is I = SCN>Cl>MES>aspartate (ASP) while the slope conductance sequence is I = SCN = Cl>MES>ASP (Table 1 & 2). The Cl_swell_ I–V relationship has a slight “S” shape, revealing rectification. An extended step protocol further illustrates features of S2R+ Cl_swell_ (Figure 1C). I_Clswell_ exhibits an initial instantaneous activation followed by a second slow activation phase, suggesting that more than one type of Cl^−^ conductance is turned on in S2R+ cells with cell swelling. In contrast, Chien & Hartzell [15] reported a single phase, time-independent I_Clswell_ activation in their S2 cells, perhaps indicating Cl^−^ channel expression differences in the two cell lines. Tail currents, normally indicating time dependence of deactivation of the channel, are evident in our recordings. However, since tail currents were not observed under symmetrical recording conditions [15], we attribute these currents to the exit of intracellular Cl^−^ accumulated during the prolonged steps. S2R+ I_Clswell_ has an interesting pharmacological profile (Figure 2A). Even at a high concentration (100 µM), the non-specific chloride channel blocker 4,4′-diisothiocyano-2,2′-stilbenedisulfonic acid (DIDS) [25] blocks less than 25% of the S2R+ I_Clswell_ (Figure 2A). 4-2(2-butyl-6,7-dichloro-2-cyclopentyl-indan-1-on-5-yl (DCPIB), a mammalian selective I_Clswell_ blocker [26], fails to completely block S2R+ I_Clswell_ at 30 µM (Figure 2A & E). DCPIB blocks in a voltage-dependent manner (Figure 2E); at 0 mV 52**%** of S2R+ I_Clswell_ is blocked, while 90**%** is blocked at 80 mV. Surprisingly, furosemide, a Na-K-2Cl cotransporter (NKCC; SLC12A2) blocker, almost completely inhibits S2R+ I_Clswell_ at 1 mM (Figure 2A & 3C).

**Table 1:** Relative Permeabilities.

	P_X_/P_Cl_					n
	I	SCN	Cl	MES	ASP	
S2R+	1.5±0.12	1.6±0.15	1	0.74±0.15	0.07±0.015	3
dBest1	1.8±0.05	2±0.04	1	0.63±0.04	0.09±0.0017	5
mBest2	1.7±0.15	2.9±0.68	1	0.45±0.14	0.05±0.006	6
d64m	2.2±0.34	2.6±0.11	1	0.43±0.14	0.07±0.011	4
HEK	1.5±0.06	1.8±0.2	1	0.62±0.005	0.11±0.003	6

**Table 2:** Slope Conductance.

	Slope Conductance (G; I/V)					n
	I	SCN	Cl	MES	ASP	
S2R+	29.6±6.8	26.9±5.9	28±7.5	16.4±3.5	7.6±1.5	3
dBest1	8.2±1.5	6.6±1.1	5.2±0.7	3.3±0.4	1.9±0.3	5
mBest2	10±4.9	2.4±0.95	6.6±3.4	2±1.3	1.7±1.1	6
d64m	10±3.5	2.5±0.36	4.4±0.3	1.9±0.5	1.7±0.3	3
HEK	26.2±8.2	24.9±7.1	26.9±7.9	9.5±5.4	5.4±2.7	6

### H148Q-YFP Reliably Reports the Activity of S2R+ Cell Cl_swell_ Channels

Cl_swell_ conducts iodide better than chloride, favoring the use of the H148Q-YFP indicator as a reporter of its activity (I^−^ K_D_ = 20 mM and Cl^−^ K_D_ = 100 mM [19], [27], [28]). Several anion-sensitive YFP variants accurately quantify intracellular Cl^−^ concentration or changes [27], [29]–[31]; anion binding near the YFP chromophore suppresses fluorescence emission by altering chromophore resonance [19]. H148Q-YFP was chosen for Cl_swell_ detection because it is bright and potently suppressed by I^−^; these properties are critical for good signal-to-noise ratios during screening. H148Q-YFP (pK_a_ = 6.7) is also sensitive to intracellular pH changes [19], [28]. S2R+ cells stably expressing H148Q-YFP maintain their fluorescence in 240 mOSM NaCl however (Figure 3A), indicating that cell swelling does not appreciably alter intracellular pH. Subsequent replacement of bath Cl^−^ with I^−^ rapidly suppresses indicator fluorescence by 50% as I^−^ enters the cells through open channels and interacts with the probe. The large fluorescence change and low intrinsic assay variability favor clear separation of potential hits. In the absence of hypo-osmotic solution, I^−^ is unable to enter the S2R+ cells and fluorescence is maintained (Figure 3B), indicating that S2R+ cells lack alternative constitutively active I^−^ entry pathways that could confound our ability to identify the Cl_swell_ channel. Further, furosemide block of open Cl_swell_ channels prevents appreciable fluorescence suppression (Figure 3C & D), suggesting that RNAi effectively targeting the Cl_swell_ channel will be readily identifiable as hits.

### Genome-wide RNAi Screening of H148Q-YFP S2R+ Cells Identifies dBest1 as the Cl_swell_ Channel

**Figure 4:**
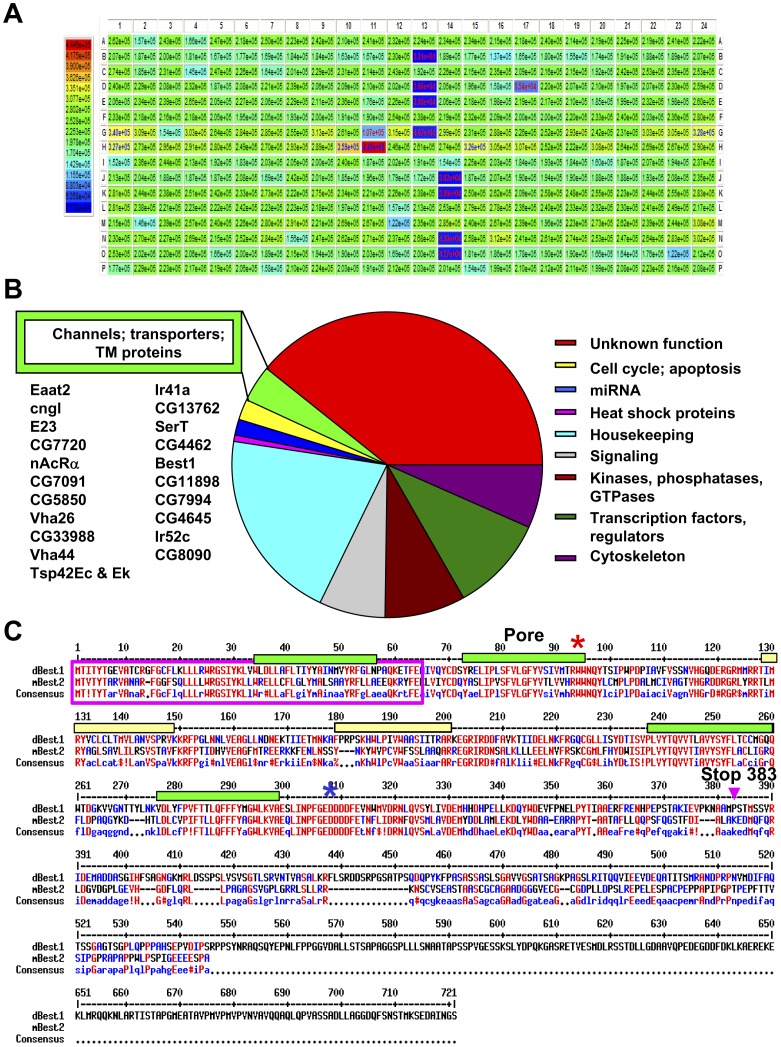
Genome-wide RNAi screening of H148Q-YFP S2R+ cells identifies Cl_swell_ channel candidates and regulators. (**A**) RNAi treatment alters S2R+ cell H148Q-YFP fluorescence levels. Heat map plate reader data following 5d RNAi treatment (240 mOSM Cl^−^). Fluorescence was subsequently measured in 240 I^−^. Wells where the I^−^ to Cl^−^fluorescence ratio was high are hits. Control RNAis are found in columns 13 and 14. Thread RNAi is in 13B, 13G, 14K, and 14N. Rho RNAi is in 13D, 13E, 14J, and 14O. GFP RNAi is in 13C, 13F, 14L, and 14M. Wells with elevated fluorescence in 240 mOSM Cl^−^ are shown in red and orange. (**B**) Functional classification of the 595 hits identified in our screen. 21 hits were transmembrane proteins of unknown function, putative ion channels, or transporters. 9 candidates with human homology were further evaluated (Table S1). (**C**) Protein sequence alignment of dBest1 and mBest2 (Multalign; multalin.toulouse.inra.fr/multalin/). Green bars indicate transmembrane domains. Yellow bars indicate other putative α-helices (SOSUI; bp.nuap.nagoya-u.ac.jp/sosui/). A red star indicates the W94C mutation. A blue star indicates the Ca^2+^-binding bowl. A pink arrow indicates Stop 383. A pink box outlines the region switched in the d64m chimera.

The primary screen was conducted at the Harvard/HHMI *Drosophila* RNAi Screening Center using our stable H148Q-YFP-expressing S2R+ cell line. Each well of sixty-six 384-well assay plates contained a dsRNA targeting 1 of 13,900 genes encoding proteins or non-coding RNAs (DRSC 2.0; Figure 4A). Five days after S2R+ cells were treated with RNAi, we assessed cellular fluorescence under swell conditions in the presence of Cl^−^ and I^−^. Wells with fluorescence or ratio (I^−^ fluorescence/Cl^−^ fluorescence) changes greater than 1.5 times the standard deviation of the plate mean were initially considered as hits (Cl_swell_ channel candidates or regulators of its activation pathway). We pared the list of 595 hits to genes with mammalian homologs and those with predicted transmembrane domains (Figure 4B, *Table S1*). In a secondary screen, we confirmed that each RNAi significantly reduced swelling-induced fluorescence and targeted only the mRNA from the identified gene (qPCR). We then directly measured I_Clswell_ via whole-cell voltage clamp. Candidates genes, whose RNAi significantly reduced the S2R+ cell I_Clswell_, were cloned and expressed in HEK293 or CHO-K1 cells. I_Clswell_ was then measured via whole-cell recording and compared with currents from untransfected cells. The only candidate of our screen to satisfy all the criteria for a Cl_swell_ channel was dBest1 (*Table S1*).

### DRSC26457 RNAi Targeting dBest1 Eliminates I_Clswell_

**Figure 5:**
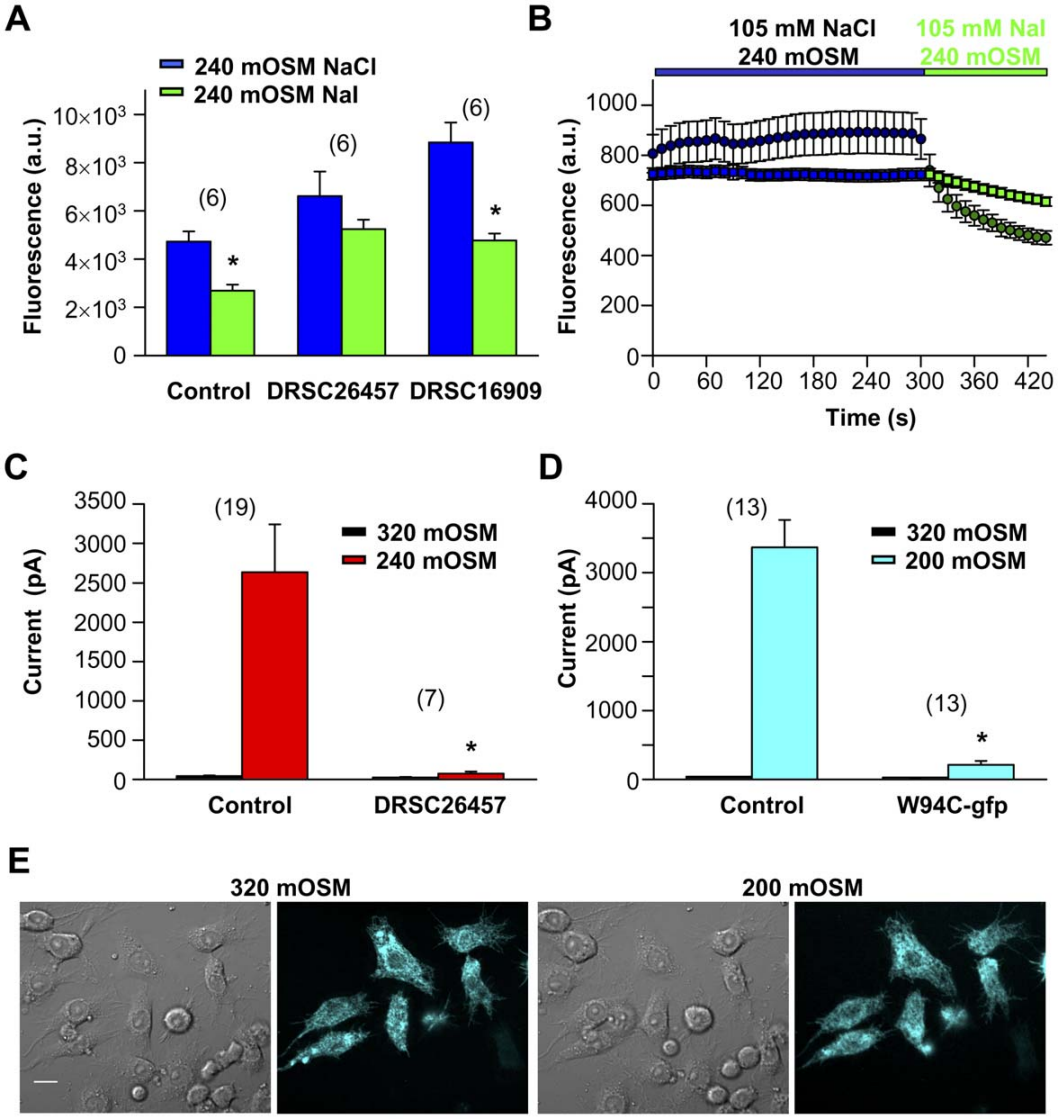
DRSC26457 identifies dBest1 as Cl_swell_. (**A & B**) RNAi efficiently targeting dBest1 prevents significant I^–^induced H148Q-YFP suppression following hypo-osmotic stimulation. Fluorescence is in arbitrary units (a.u.). (**A**) Plate reader assay. DRSC26457 RNAi treatment resulted in a fluorescence decrease of 6.4% ±19. Control and DRSC16909 RNAi treatment resulted in fluorescence decreases of 40.5% ±9.1 and 43.7% ±6.2 respectively. * 240 mOSM NaCl and NaI fluorescence levels are significantly different (Student’s t-test, p<0.05). (**B**) Imaging assay. The fluorescence levels of individual S2R+ cells treated with control or dBest1 DRSC26457 RNAi were measured during hypo-osmotic stimulation in the sequential presence of Cl^−^ and I^−^. The fluorescence of control cells decreased by 56% (n = 44); in contrast, the fluorescence of dBest1 DRSC26457 RNAi treated cells was suppressed by 15% (n = 174). (**C**) dBest1 DRSC26457 RNAi eliminated I_Clswell_ in S2R+ cells. Following dBest1 RNAi treatment I_320_ is not significantly different from I_240_ (Student’s t-test, p = 0.1). * control and RNAi treated I_240_ are significantly different (Student’s t-test, p = 0.02). (**D**) dBest1 W94C-gfp overexpression suppresses S2R+ I_Clswell_. * control and W94C-gfp I_200 mOSM_ are significantly different (Student’s t-test, p = 0.02). (**E**) Confocal images of dBest1 W94C-gfp overexpression in S2R+ cells. Images were obtained before (320 mOSM) and after swell (200 mOSM). Scale bar indicates 10 µm.

dBest1 is a protein of 769 amino acids containing 4 transmembrane domains [32], [33] (Figure 4C). It is one of four Bestrophin family members in *Drosophila,* with highest homology to mBest2/hBest2 (51% identity and 67% similarity; BLAST). Hartzell and colleagues first proposed that dBest1 was a chloride channel activated by high intracellular Ca^2+^ and cell swelling [15], [16]. In our H148Q-YFP fluorescence assay dBest1 RNAi DRSC26457 abrogated the fluorescence change normally observed when I^−^ enters the S2R+ cells through activated Cl_swell_ conductances (Figure 5A, B). Interestingly, DRSC26457 also decreased the baseline fluorescence variability of S2R+ cells (Figure 5B), suggesting that I_dBest1_ contributes to resting intracellular Cl^−^ concentrations. S2R+ I_Clswell_ was essentially eliminated by dBest1 RNAi DRSC26457 treatment (Figure 5C). This RNAi specifically and effectively reduced dBest1 mRNA by 91.5% ±0.5 (n = 3; qPCR); mRNA levels for the 3 remaining Bestrophin members and other Cl_swell_ candidates were unaffected. A second RNAi targeting dBest1 (DRSC16909; corresponds with dB1S [15]) was part of our initial screen. It was less effective at knocking down dBest1 mRNA (85% reduction, n = 3; significantly less than DRSC26457; p<0.001, Student’s t-test) and had two predicted off-target hits: CG4623 (20/20) and CG16711 (18/18). DRSC16909 did not significantly alter H148Q-YFP I^–^induced fluorescence suppression (Figure 5A), and was not a hit in our initial screen. It is possible that the 15% remaining mRNA translated sufficient amounts of functional dBest1/Cl_swell_ channels to exclude it as a hit in our screen. This prospect emphasizes the importance of validated, effective RNAi for accurate screening.

### Mutant dBest1 W94C Significantly Reduces S2R+ I_Clswell_

To substantiate the conclusion that dBest1 is an essential component of the Cl_swell_ channel, we tested whether a mutant dBest1 would act as a dominant negative regulator of I_Clswell_. In humans, Bestrophin 1 is mutant in vitelliform macular dystrophy (Best’s disease [34], [35]). One mutation, W93C, occurs in a conserved sequence of the channel’s putative pore [36], [37] (Figure 4C). When we expressed the homolog dBest1 W94C-gfp in S2R+ cells, I_Clswell_ was significantly reduced (Figure 5D). Interestingly, the late activating component of I_Clswell_ remained clearly evident at depolarized potentials (Figure S1). We could not study this current in more detail as a loss of cell membrane integrity rapidly ensued. We conclude that dBest1 is responsible for the early activating S2R+ cell I_Clswell_. W94C might interact with WT dBest1 to disrupt the Cl_swell_ channel pore or it may prevent proper protein trafficking [38]. In S2R+ cells, dBest1 W94C-gfp has a distinct intracellular expression pattern unaltered by osmotic changes (Figure 5E), suggesting the latter explanation over the former. Regardless, dBest1 W94C has a dominant negative impact on I_Clswell_, further evidence that dBest1 is integral to the Cl_swell_ channel.

Another disease-associated Bestrophin mutation, D308A, occurs in a putative Ca^2+^ -binding bowl located in the channel’s C-terminus (Figure 4C; blue star). D308A is proposed to eliminate Bestrophin activation by disruption of calcium binding [39]. We introduced this mutation into dBest1 to determine if activation by calcium and cell swelling could be separated. Unfortunately dBest1 D308A-gfp was not functional in HEK cells (data not shown). Three possible explanations may underlie this result: 1) activation by multi-modal stimuli is simultaneously disrupted by the mutation; 2) the mutation causes protein misfolding and the channel function has been eliminated for reasons unrelated to activation; 3) the mutant channel is mislocalized. Our GFP-tagged protein was expressed (data not shown), but we cannot exclude the possibility that it mislocalizes or fails to interact appropriately with other proteins necessary for I_Clswell_ activation or channel function [34].

### Exogenous dBest1 Expression Creates a *Drosophila*-like I_Clswell_

**Figure 6:**
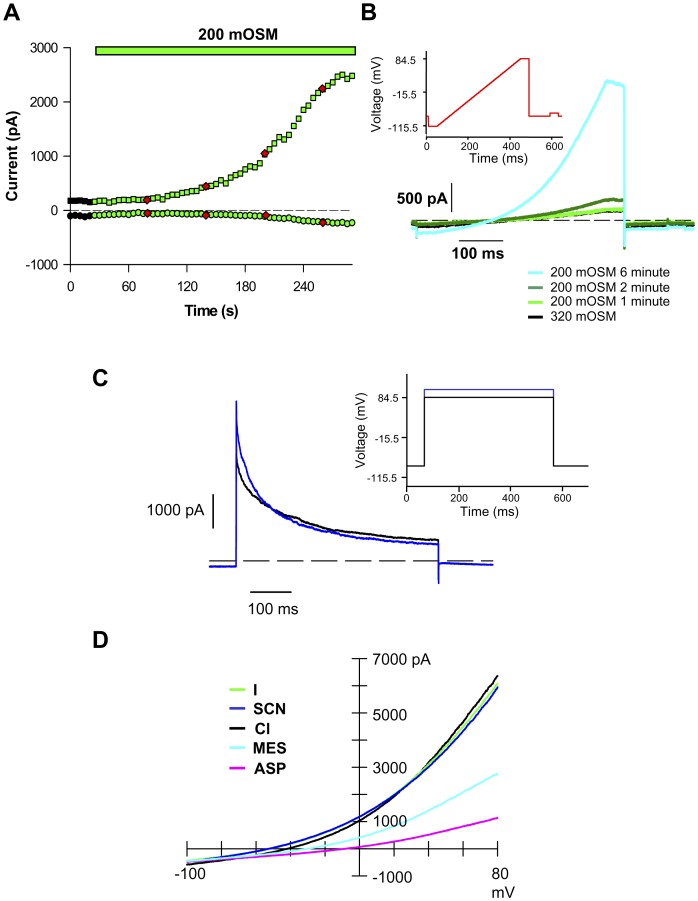
The endogenous HEK cell I_Clswell_ has characteristic mammalian I_Clswell_ properties. (**A**) HEK cell I_Clswell_ develops slowly, reaching steady state after 5 min exposure to 200 mOSM solution. ⧫ indicate min in 200 mOSM. (**B**) Ramp protocol (inset) assessment of the HEK I_Clswell_. Little I_Clswell_ has developed after 2 min in 200 mOSM solution. The steady state I_Clswell_ is outwardly rectifying and inactivating at positive potentials. No tail currents are apparent. (**C**) Step protocol (inset) assessment of the HEK I_Clswell_. Rapid inactivation is observed at positive potentials. No tail currents are apparent. (**D**) Relative permeability and slope conductance sequences for the endogenous HEK I_Clswell_ are SCN = I>Cl>>MES>ASP vs SCN = I = Cl>>MES>ASP.

Exogenous expression of a candidate protein substantiates whether the protein is necessary and/or sufficient in a given process. Our secondary screen assessed whether candidate protein expression resulted in a novel I_Clswell_ or augmented the endogenous HEK I_Clswell_ (*Table S1*). The HEK cell line chosen for candidate over-expression lacked constitutive I_Cl_ and I_SCN_ (potentially contaminating conductances attributable to SLC1A family member expression [40]; data not shown). The endogenous HEK I_Clswell_ develops very slowly (Figure 6A & B); a two fold increase was noted within the first 2 min of swell. Once the HEK I_Clswell_ reaches steady state, however, it has increased more than forty fold (44.4±10.7 fold, n = 29; Student’s t-test, p<0.000005). Tail currents are absent (Figure 6B & C). Characteristic voltage-dependent inactivation develops during steps to positive potentials (Figure 6C). HEK I_Clswell_ is anion selective; its permeability and conductance sequences match closely to those of S2R+ I_Clswell_ (Figure 6D; Table 1 & 2). The HEK I_Clswell_ pharmacological profile (Figure 2C) correlates well with the literature. 100 µM DIDS, slightly above the reported IC_50_ [41], blocks 78% of the HEK I_Clswell_ at +80 mV (Figure 2C). DCPIB has an IC_50_ of 4 µM [26]; at 30 µM 100% of HEK I_Clswell_ is blocked (Figure 2C). 1 mM furosemide barely inhibits HEK I_Clswell_ (Figure 2C). The endogenous HEK I_Clswell_ recapitulates the key features noted for the mammalian I_Clswell_ [1].

**Figure 7:**
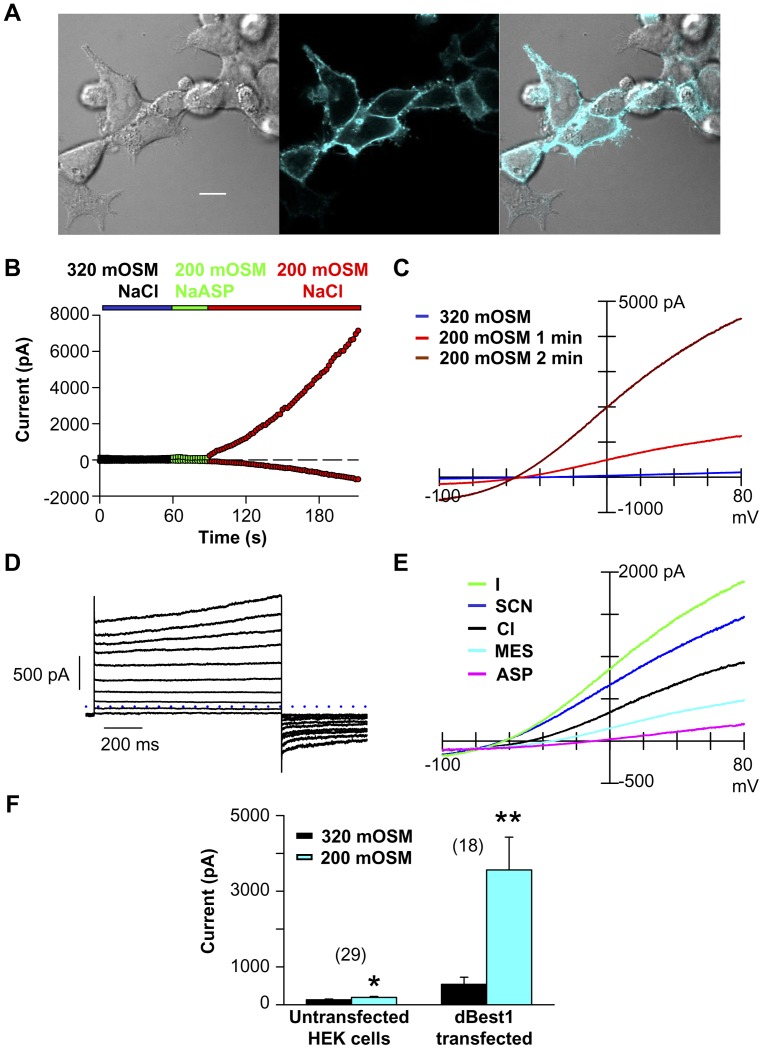
dBest1 overexpression in HEK cells produces a S2R+ cell-like I_Clswell_. (**A**) dBest1-gfp targets to the membrane of HEK cells. Confocal images of dBest1-gfp overexpressed in HEK-293 cells. The DIC image is on the left, GFP in the middle, overlapped images on the right. Scale bar indicates 10 µm. (**B**) I_dBest1_ rapidly develops within the first 2 min of hypo-osmotic stimulation. (**C**) The developing I_dBest1_ has the same “S” shape rectification as the endogenous S2R+ cell I_Clswell_. (**D**) Step protocol shows that I_dBest1_ shares time-dependent activation and tail current properties with S2R+ cell I_Clswell_. (**E**) The constitutively active I_dBest1_ and S2R+ cell I_Clswell_ selectivity sequences are very similar. (**F**) I_dBest1_ is clearly separable from the endogenous HEK cell I_Clswell_. I_dBest1_ increases 15.8 fold ±4.5 (n = 18; ** paired Student’s t-test, p<0.005) in the first 2 min of hypo-osmotic stimulation; the endogenous HEK cell I_Clswell_ increases 2.1 fold ±0.4 (n = 29; * paired Student’s t-test, p<0.05).

Bestrophin proteins are not universally accepted as *bona fide* chloride channels; alternatively they are intracellular ion channel regulators [33], [42], [43]. dBest1-gfp is clearly observed on or near the surface of HEK-293 cells (Figure 7A). Its expression results in a *Drosophila*-like I_Clswell_ (Figure 7B–E). Constitutively active I_dBest1_ is apparent in iso-osmotic 320 mOSM solution and is significantly increased 16±4.5 fold (Student’s t-test, p<0.005) during the first 2 min of hypo-osmotic stimulation (Figure 7B). I_dBest1_ has the same “S”-shaped rectification as *Drosophila* I_Clswell_ during ramps (Figure 7C); tail currents and time-dependent activation are both apparent in the step protocol (Figure 7d). I_dBest1_ is anion selective; it has the same permeability and conductance sequences as S2R+ I_Clswell_ and HEK I_Clswell_ (Figure 7E; Table 1 & 2). Strikingly, I_dBest1_ and the endogenous S2R+ I_Clswell_ share a similar pharmacological profile that differs significantly from HEK I_Clswell_ (Figure 2A–C). 100 µM DIDS inhibits 35% of I_dBest_, while 30 µM DCPIB blocks 45%. 1 mM furosemide blocks nearly 100% of the I_dBest1_. We conclude that dBest1 expression results in a *Drosophila*-like I_Clswell_; it cannot be attributed to endogenous HEK I_Clswell_ upregulation.

### dBest1 Swell Activation can be Conferred on the Swell-insensitive mBest2

**Figure 8:**
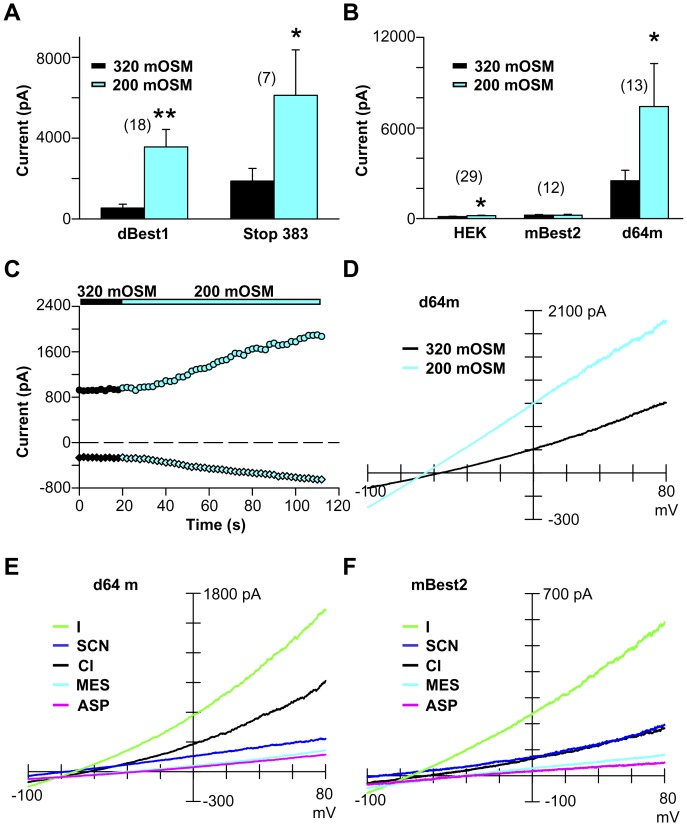
Chimeras between dBest1 and mBest2 confer swell activation on mBest2. (**A**) Truncation of dBest1 does not interfere with swell sensitivity. Constitutive currents are apparent with both dBest1 and Stop 383 overexpression in iso-osmotic solutions (320 mOSM). With swell, current increased dramatically for both constructs (Student’s t-test, * p<0.05, ** p<0.005). (**B**) d94m current increases significantly within the first 2 min of swelling (* Student’s t-test, p<0.05). (**C**) Time course for d64m swell activation. (**D**) Current-voltage relations for constitutive d64m, and following 2 min 200 mOSM solution. (**E**) Selectivity sequence for the constitutively active d64m current. (**F**) Selectivity sequence for the constitutively active mBest2 currents.

The structural domains necessary for swell-induced channel activation are unknown. Although dBest1 has a long poorly conserved C-terminus (Figure 4C), it is not necessary for swell activation. dBest1 remains swell-sensitive despite the removal of up to 338 of its C-terminal amino acid residues (Stop 383, Figure 4C; Figure 8A). Next we examined whether chimeras might reveal the domains underlying swell activation. dBest1’s closest mammalian homolog, mBest2, is not activated by hypo-osmotic solutions (Figure 8B). Chimera d64m (the first 64 residues are dBest1; the remaining residues are identical to those of mBest2; Figure 4C) expression resulted in a constitutively active current that more than doubled with swelling (2.3 fold ±0.3 increase; Student’s t-test, p<0.05; Figure 8B–D). The d64m chimera maintained the relative permeability and slope conductance of mBest2 (Figure 8E & F; Table 1 & 2), suggesting that the channel’s pore domain is downstream of residue 64. Two other groups have assessed mBest2 selectivity [44], [45] and found greater permeability for SCN than we report here. Both groups used high intracellular calcium to activate I_mBest2_; we report constitutive I_mBest2_ measured with high internal calcium buffering (i.e. <10 nM free calcium). Our HEK cell line was also screened for potentially contaminating I_SCN_ (data not shown) attributable to SLC1A family member expression [40]. The pharmacological profile of I_d64m_ noticeably diverged from both that of HEK I_Clswell_ and I_dBest1_ (Figure 2). Furosemide blocked 75% of I_d64m_, while DIDS and DCPIB both blocked I_d64m_ to near completion (Figure 2D). We conclude that the dBest1 N-terminal domain is required for swell activation of the mBest2 channel. The reverse chimera (m64d) was nonfunctional; exogenous currents were not observed with swelling or in the presence of high intracellular Ca^2+^ (data not shown). We cannot conclude with this data however, that the N-terminus is a “swelling” domain as it lacks any predictive motifs. We hypothesize that it works in concert with domains present both in dBest1 and mBest2 to facilitate swell activation. The strong correlation between S2R+ I_Clswell_ and I_dBest1_, combined with the unique selectivity and pharmacology of the d64m chimera, support the conclusion that the Bestrophin protein itself forms the Cl_swell_ channel rather than functioning as an auxiliary subunit.

## Discussion

Our study validates the H148Q-YFP fluorophore as a reliable reporter of Cl_swell_ channel activity in genome-wide RNAi screening studies. H148Q-YFP has been employed very effectively in the identification of novel chloride channel activators, modulators, and blockers of CFTR and Ca^2+^-activated Cl^−^ channels [18]. This is the first reported RNAi screen using an anion-sensitive fluorescent protein to assign molecular identity to a chloride channel. Our screen supports the findings of the Hartzell lab [15]: dBest1 RNAi eliminates *Drosophila* I_Clswell_.

We found that the RNAi effectiveness was essential for Cl_swell_ candidate identification. Two separate dBest1-targeting RNAi’s were part of our initial screen: DRSC26457 and DRSC16909 (which corresponds to dB1S [15]), but only DRSC26457 was a hit. qPCR reported a 95% reduction in dBest1 mRNA with DRSC26457 treatment versus an 85% reduction with DRSC16909. Hartzell and colleagues found that S2 cell I_Clswell_ was significantly reduced following treatment with 0.4 µg of DRSC16909 [15], while in our screen each assay well had a standardized 0.25 µg of RNAi. Using more RNAi may have effected greater target knockdown and resulted in the detection of DRSC16909 as a hit in our screen. This result emphasizes the importance of RNAi effectiveness in hit identification.

Exogenously expressed I_dBest1_ and endogenous S2R+ I_Clswell_ share similar characteristics, including time-dependent activation, tail currents, relative permeability sequences, slope conductance sequences, and pharmacological profiles. The shared properties of *Drosophila* I_Clswell_ and I_dBest1_ suggest that the same protein forms the channel responsible for both. Bestrophin is a known ion channel modulator, altering voltage-gated calcium channel activity [46]. If dBest1 expression simply modulated or upregulated the endogenous HEK Cl_swell_ channel expression, we would have expected the resulting I_Clswell_ to maintain the properties of HEK I_Clswell_. Instead we observed that dBest1 introduced an exogenous *Drosophila*-like I_Clswell_ whose development preceeded that of the endogenous HEK I_Clswell_. I_dBest1_ matched the pharmacological profile of the S2R+ I_Clswell_. Moreover, we found that the exogenous *Drosophila*-like I_Clswell_ permeability and conductance sequence could be transformed into that of mBest2 with the d64m chimera. The pharmacological profile of I_d64m_ was again significantly different from the endogenous HEK I_Clswell_. We conclude that dBest1 is the *Drosophila* Cl_swell_ channel.

Several Bestrophin mutations are associated with vitelliform macular dystrophy [36], [37]. How these mutations are causally linked to the disease is not clear. Here we found that overexpression of the disease-linked W94C dBest1 mutant in S2R+ cells significantly suppressed the endogenous *Drosophila* I_Clswell_. The W94C mutation occurs in the putative pore of dBest1 and thus may disrupt Cl_swell_ conductance. However, the fluorescently tagged W94C dBest1 protein appears to localize to intracellular compartments, consistent with mislocalization. Milenkovic *et al*, have recently proposed that disease-associated Bestrophin mutations cause defects in intracellular trafficking [38]. Both scenarios may explain the dominant negative effect of dBest1 W94C on *Drosophila* I_Clswell_: non-functional, pore-disrupting, mutant Bestrophin proteins complexing with wild-type dBest1 may be largely retained within the endoplasmic reticulum. The end result would be the elimination of endogenous *Drosophila* I_Clswell_. Our experiments support the hypothesis that mutant Bestrophin W93C expression could significantly disrupt chloride flux and homeostasis in the human macula, contributing to the disease state.

The distinction of Bestrophin function in *Drosophila* versus mammalian cells is most clearly illustrated by Hartzell and colleagues [16]. I_Clswell_ measured in peritoneal mast cells isolated from *mBest1*_−/−_, *mBest2*_−/−_, and *mBest*1/2 double knockout mice was identical to *wild-type* I_Clswell_. hBest1 and mBest2 are swell sensitive in that their currents are inhibited by hyperosmotic solutions. However, their activity does not increase with swell [12]. We confirm here that mBest2 activation does not increase when cells swell. Our d64m chimera contained only a small portion of dBest1, yet it responded to cellular swelling. The crucial N-terminal region contains no distinct association domains or predictive structures that might explain its coupling to changes in cell stretch, tension, or osmolality. We speculate that the N-terminus contributes to a required tertiary structure that enables swell signaling events to activate the dBest1 channel.

Our genome-wide RNAi screen of S2R+ cells and follow-up study firmly establishes that the dBest1 protein forms the *Drosophila* Cl_swell_ channel. It further validates a live cell genetically engineered fluorescent screening platform to identify other mammalian chloride channels.

## Materials and Methods

### Generation of the S2R+ YFP- H148Q Stable Cell Line

The stable S2R+ cell line expressing a halide-sensitive YFP (H148Q-YFP; kindly provided by Dr. Alan Verkman, UCSF) was generated with a selection vector (pCoBlast). H148Q-YFP was subcloned into the pAc5.1/V5-HisA vector (Invitrogen, CA). The S2R+ cells were transfected by electroporation (Amaxa cell line nucleofector kit V; Lonza). Cells were placed under selective pressure with 25 µg/ml blasticidin for 2 weeks. Two rounds of fluorescence-activated cell sorting (FACS; DFCI Flow Cytometry Core Facility) normalized YFP fluorescence intensities. These S2R+ cells exhibited a robust I_Clswell_ as described in Figure 1. S2R+ cells were maintained in Schneider’s *Drosophila* medium (Invitrogen), with 10% heat inactivated fetal bovine serum (Invitrogen), and 1% penicillin/streptomycin (Sigma-Aldrich). For the primary screen, cells were spun down and resuspended in serum-free medium at a density of 9×10^4^ cells/ml. 10 µl of the cell suspension was added to each of the 384 wells using the Matrix Wellmate 8-channel microplate dispenser (ThermoScientific; DRSC). Cells were incubated for 30 min, then 30 µl of serum containing medium were added to each well. Cells were cultured for 5 d before the swell assay was performed (5 d RNAi treatment is necessary to sufficiently knock down proteins with slow turnover rates). For the secondary screen, cells were plated at a density of 40% in a 6 well dish. Once cells were adherent, the medium was replaced with 1 ml of serum-free medium containing 0.015 µg/µl dsRNA. Cells were incubated for 30 min at room temperature followed by addition of 3 ml of serum-containing medium to each well. Transfections were performed in duplicate; 1 well was used for functional studies and the other for qPCR analysis of knockdown at day 5.

### Screening Solutions

Iso-osmotic (320 milliOsm/kg; mOSM) solutions contained in mM: 105 NaCl or NaI, 2 CaCl_2_, 1 MgCl_2_, 5 KCl, 10 HEPES, 10 Glucose, 90 Mannitol (pH 7.4 NaOH). For hypo-osmotic (240 mOSM) solutions, mannitol was omitted. 1 mM furosemide (Sigma-Aldrich) blocked 100% of the S2R+ I_Clswell_ and prevented significant H148Q-YFP suppression. Furosemide is a specific blocker of Na-K-2Cl co-transporters (SLC12A2) at concentrations in the µM range.

### The Primary Screen

Our genome-wide screen was conducted at the Harvard/HHMI *Drosophila* RNAi Screening Center using the DRSC 2.0 Genomewide RNAi Library. DRSC 2.0 is a collection of dsRNAs for genome-wide RNAi knockdown covering ∼ 13,900 genes encoding proteins and non-coding RNAs while minimizing off-target effects due to sequence similarity to other genes. Each gene is targeted by 1.3 dsRNA/gene. The screen consisted of 66 384-well assay plates in duplicate. Each well of the 384-well plate contained 5 µl of 0.05 µg/µl dsRNA in water (0.25 µg dsRNA/well). Each plate contained control RNAi specific for *Thread* (*Drosophila* inhibitor of apoptosis protein), *Rho* (a small GTPase activator of the EGFR signaling pathway), and *GFP*. On day 5 of RNAi treatment, the cellular fluorescence of the H148Q-YFP probe was measured under several treatment conditions using the Analyst GT plate reader (Molecular Devices; DRSC). The probe was excited at 485 nm and emissions collected at 530 nm. Before fluorescence measurements were taken, the media was aspirated (384-well aspirator; VP Scientific) and the cells were equilibrated in 80 µl of 320 mOSM NaCl solution. After 10 min this solution was removed by aspiration and cells incubated in 240 mOSM NaCl for 5 min. Fluorescence was then measured, the NaCl solution removed and cells were incubated in 240 mOSM NaI for 5 min. Fluorescence was again measured; the change in fluorescence was determined by dividing the fluorescence in 240 mOSM NaI by that in 240 mOSM NaCl. Wells with fluorescence or ratio changes (240 mOSM I^−^ fluorescence/240 mOSM Cl^−^ fluorescence) greater than 1.5 times the standard deviation (1.5×S.D.) of the plate mean were initially considered as hits (candidates of Cl_swell_ channel or regulators of its activation pathway). False positives could potentially result if the RNAi treatment caused a high internal pH as H148Q-YFP has a pKa of 6.7. Cell death was detected in control wells indicating effective RNAi treatment.

### Generation of dsRNA

cDNA templates were generated by PCR amplification of genomic DNA using primers designed by the DRSC (SnapDragon tool). These primers had the T7 promoter sequence (TAATACGACTCACTATAGGG) added to the 5' end of both primers. The templates generally corresponded to exons but occasionally sequences with two or more exons interrupted by introns were used. The PCR fragments were ∼150–600 base pairs in length, and any complete 19-mer homology to other genes that could lead to non-specific dsRNA are reported. Individual RNAi sequences used here are found in the DRSC website (www.flyrnai.org). dsRNAs against *Drosophila* were synthesized with the MEGAscript in vitro transcription kit (Ambion). RNA was purified with the RNeasyPlus mini kit (Qiagen) and stored at −80°C.

### qPCR Analysis of RNAi Efficiency

After 5 d RNAi treatment, RNA was prepared from the S2R+ cells using the RNeasy Plus mini kit (Qiagen). 2.5 µg RNA was used for each first-strand cDNA synthesis reaction (SuperScript Vilo cDNA Synthesis kit, Invitrogen). Primers for qPCR were designed on the NCBI/PrimerBlast site (http://www.ncbi.nlm. nih.gov/tools/primer-blast/) with the following restrictions: PCR product size was between 70 and 300 bp, primer melting temperatures were between 57 and 63°C, primers spanned an exon-exon junction, and primers were specific to the intended PCR template as determined by BLAST analysis of the *Drosophila melanogaster* Refseq mRNA database. Primer sets were only used if the melting curve had a single peak. The RT^2^ Real-Time SYBR Green/Rox PCR master mix (SABiosciences) was used for qPCR. qPCR reactions were set up in quadruplicate to minimize pipetting errors, and run on the Mastercycler ep Realplex real-time PCR system (Eppendorf). Average cycle numbers for each primer set were normalized to either dTaf8 or dAct79b average cycle numbers.

### Secondary Screening of Candidates

Comprehensive bioinformatics analysis of the hit list was performed to identify potential candidates for Cl_swell_. Hits were limited to those with human homologs and at least a single transmembrane domain. Potential regulators of the Cl_swell_ activation pathway were left for future consideration. The effects of RNAi on fluorescence changes were confirmed by plate reader or imaging experiments. The specificity and effectiveness of the RNAi was assessed by qPCR. S2R+ cells treated with RNAi were patch clamped and I_Clswell_ was directly measured. Candidates were cloned and expressed in HEK293 or CHO-K1 cells. I_Clswell_ was measured by whole-cell patch clamp recording.

### Electrophysiology

Whole-cell patch clamp recordings were made at room temperature. Recordings were obtained using an Axopatch 200B amplifier, Digidata 1322A analog-to-digital converter, and pClamp 8.01 software (Molecular Devices, Union City, CA). Data were low-pass filtered at 2 kHz and digitized at 5 kHz. Fire-polished thin or thick wall borosilicate glass pipettes of 3–4 MΩ resistances were used for recordings; access resistance was compensated to >80%. Cells were held at −70 mV to clearly eliminate cells with leaky seals and voltage ramps (400 ms in duration) from −100 to +100 mV were applied every 2–5 s. Liquid junction potentials were corrected during analysis, and ramp data were plotted between –100 and +80 mV.

### Recording Solutions

Internal pipette solution contained (in mM): 160 CsASP, 10 Cs4BAPTA, 4 MgATP, 2 MgCl_2_, 8 NaCl, and 10 HEPES (pH 7.4 with CsOH). 10 mM BAPTA was used to prevent activation of channels by calcium and to reduce the endogenous HEK cell I_Clswell_, which is optimally activated with 100 nM Ca_i_ [47]. 240 mOSM solution composition is detailed in ‘Screening solutions’. 200 mOSM solutions contained in mM: 82.5 NaCl, NaI, NaSCN, NaMES, or NaASP, 2 CaCl_2_, 1 MgCl_2_, 5 KCl, 10 HEPES, and 10 Glucose (pH 7.4 with NaOH). 90 mM mannitol was added to bring osmolality to 320 mOSM. The relative permeabilities were estimated from the Goldman-Hodgkin-Katz equation. For our calculations, the [Cl]_i_ was set to 0 mM. Cells were held at -70 mV during all recordings, rapidly depleting Cl_i_. Cation permeability was essentially nil, as replacement of 200 mOSM NaCl solution with 200 mOSM NMDG-Cl solution did not change the reversal potential (E_rev_; data not shown). Slope conductances were calculated for each anionic substitution between the E_rev_ and +80 mV. For the S2R+ and HEK cells E_rev_ and I_+80 mV_ were measured in 200 mOSM solutions. For exogenously expressed dBest1, mBest2, and d64m E_rev_ and I_+80 mV_ were measured in 320 mOSM solutions to prevent contamination with the endogenous HEK cell I_Clswell_. The HEK cell line chosen for over-expression studies had no constitutive I_Cl_ (I_320 mOSM_ did not change when switching between Cl and ASP), the endogenous I_Clswell_ developed very slowly, and the cells did not have an endogenous I_SCN_ (attributable to SLC1A family member expression [40].

### Pharmacology

Stock solutions of DIDS (0.1 M in DMSO; Sigma), DCPIB (50 mM in EtOH; Tocris), and furosemide (1M in DMSO; Sigma) were prepared and diluted in 320 mOSM or 200 mOSM NaCl solution to their final concentrations.

### Molecular Biology

*dBest1* was a kind gift from Dr. Criss Hartzell (Emory University). All other constructs were either ordered from Open Biosystems or cloned from a *Drosophila* cDNA library or Human Brain (whole Marathon ready cDNA library; BD Biosciences). Candidate cDNAs were subcloned into pEGFP-N3 (C-terminal tag; BD Biosciences) and an engineered Red pTracer vector (untagged). We found that an N-terminal EGFP tag rendered dBest1 nonfunctional (data not shown). Using site-directed mutagenesis we introduced the W94C mutation into *dBest1* pEGFP-N3 (GeneArt site-directed mutagenesis, Invitrogen, CA). *dBest1* W94C-gfp was then subcloned into the pAc5.1 V5-HisA vector (Invitrogen, CA).

## Supporting Information

Figure S1 **The late activating component of S2R+ I_Clswell_ remains despite overexpression of dBest1 W94C-gfp.** (**A**) The late activating component of S2R+ I_Clswell_ is isolated after dominant negative elimination of I_dBest1_. Inset: Step protocol. (**B**) The late activating component of S2R+ I_Clswell_ is sharply rectifying (ramp protocol; inset). Red trace is 320 mOSM solution, blue trace is 80 s after the 200 mOSM solution change. The late activating I_Clswell_ develops after 36 s in 200 mOSM solution.
(TIF)

Table S1
**Secondary screening identifies Best1 as the** ***Drosophila*** **Cl_swell_ channel.** Candidates with transmembrane domains and human homologs were further studied to determine if they formed the Cl_swell_ channel. ✓ indicates a positive secondary screening result; X indicates a negative result. 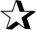 indicates that several qPCR primer sets consistently had more than 1 melting point peak suggesting nonspecific primer binding. The effectiveness of RNAi knockdown, therefore, could not be determined by qPCR. ?? indicates that two cells overexpressing SLC1A2 had substantial I_SCN-_ currents but small I_Clswell_. Thus, SLC1A2 overexpression may upregulate endogenous HEK cell I_Clswell_ in the majority of the population but does not form the channel itself. HeLa cells treated with SLC1A3 siRNA (which reduced SLC1A2 and SLC1A3 mRNA by 90% and 92% respectively) had unaltered I_Clswell_ (data not shown).(TIF)
