# Supplementary figures and images for: Anion-Sensitive Fluorophore Identifies the Drosophila Swell-Activated Chloride Channel in a Genome-Wide RNA Interference Screen
Source: PLoS One. 2012 Oct 4;7(10):e46865. doi: 10.1371/journal.pone.0046865 (PMC3464265; doi:10.1371/journal.pone.0046865)

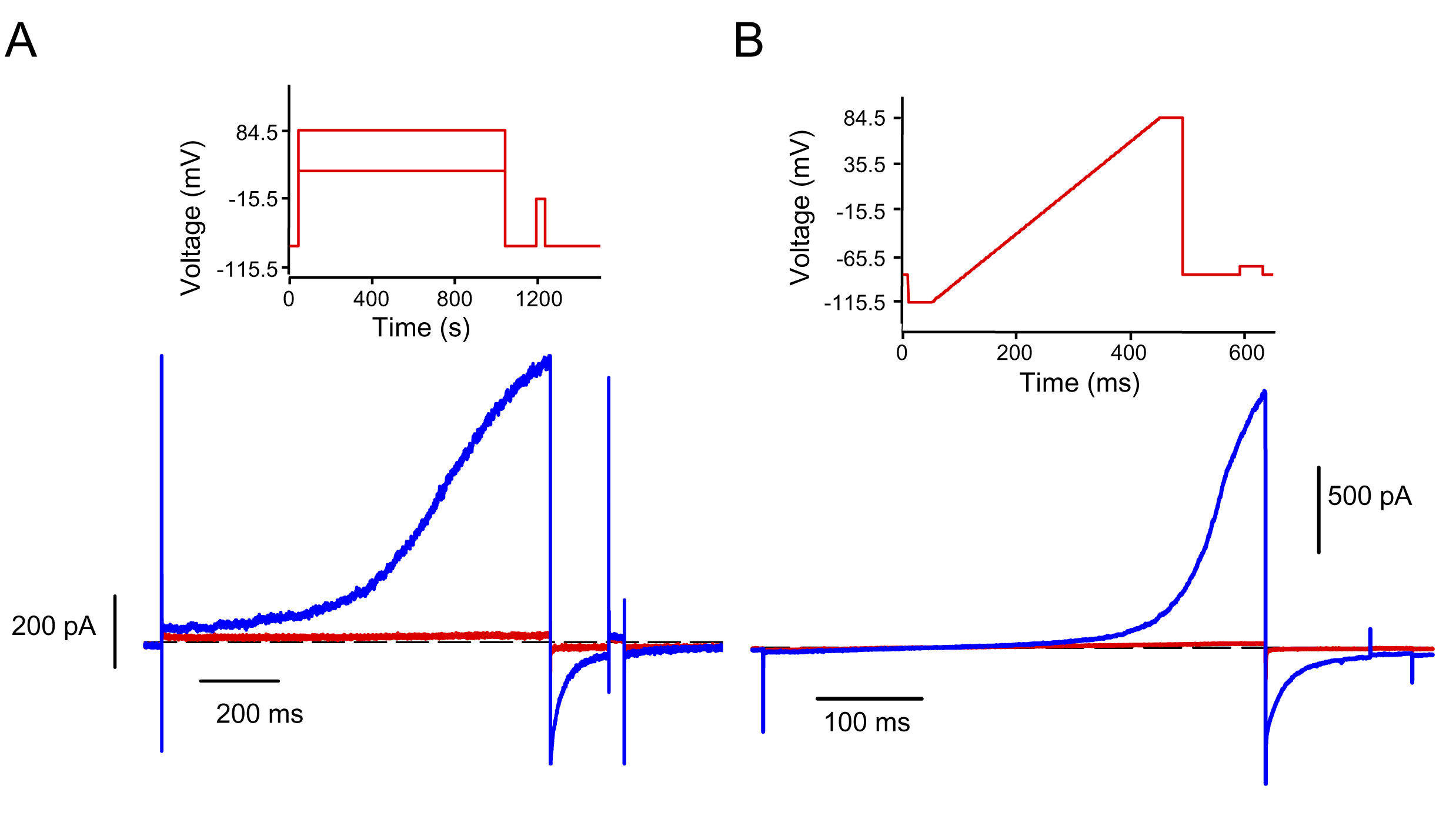

Supplement: Figure S1 — The late activating component of S2R+ IClswell remains despite overexpression of dBest1 W94C-gfp. (A) The late activating component of S2R+ IClswell is isolated after dominant negative elimination of IdBest1. Inset: Step protocol. (B) The late activating component of S2R+ IClswell is sharply rectifying (ramp protocol; inset). Red trace is 320 mOSM solution, blue trace is 80 s after the 200 mOSM solution change. The late activating IClswell develops after 36 s in 200 mOSM solution. (TIF) [file pone.0046865.s001.tif]

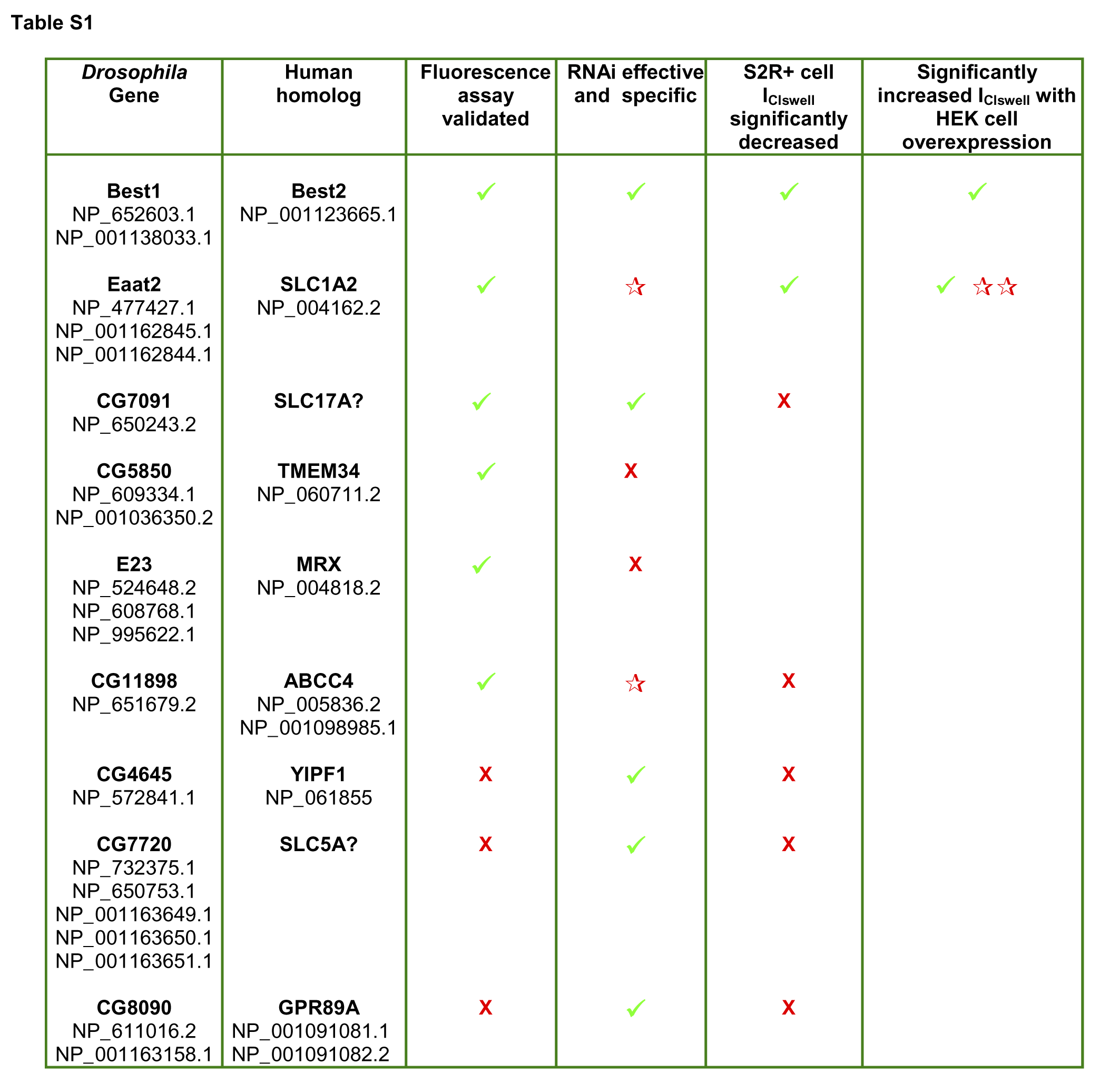

Supplement: Table S1 — Secondary screening identifies Best1 as the Drosophila Clswell channel. Candidates with transmembrane domains and human homologs were further studied to determine if they formed the Clswell channel. ✓ indicates a positive secondary screening result; X indicates a negative result. indicates that several qPCR primer sets consistently had more than 1 melting point peak suggesting nonspecific primer binding. The effectiveness of RNAi knockdown, therefore, could not be determined by qPCR. ?? indicates that two cells overexpressing SLC1A2 had substantial ISCN- currents but small IClswell. Thus, SLC1A2 overexpression may upregulate endogenous HEK cell IClswell in the majority of the population but does not form the channel itself. HeLa cells treated with SLC1A3 siRNA (which reduced SLC1A2 and SLC1A3 mRNA by 90% and 92% respectively) had unaltered IClswell (data not shown). (TIF) [file pone.0046865.s002.tif]
